# Management of External Invasive Cervical Resorption Tooth with Mineral Trioxide Aggregate: A Case Report

**DOI:** 10.1155/2013/139801

**Published:** 2013-02-13

**Authors:** Anuja Ikhar, Nikita Thakur, Aditya Patel, Rohan Bhede, Pranav Patil, Surbhi Gupta

**Affiliations:** ^1^Department of Conservative Dentistry and Endodontics, Sharad Pawar Dental College, Wardha, Maharashtra 442 004, India; ^2^Department of Conservative Dentistry and Endodontics, Bharati Vidyapeeth Dental College and Hospital, Sangli, Maharashtra 414 414, India; ^3^Department of Conservative Dentistry and Endodontics, Karmaveer Bhausaheb Hiray Dental College, Maharashtra 422 003, Nashik, India

## Abstract

Invasive cervical resorption is entirely uncommon entities and the etiology is poorly understood. A 19 year old patient presented with fractured upper left central incisor and sinus tract opening on the distobuccal aspect in cervical region. Radiographic examination shows irregular radiolucency over the coronal one-third and it extended externally towards the external invasive resorption. After sectional obturation, the defect was accessed surgically. The resorption area was chemomechanically debrided using irrigant solution. Fibre post placement using flowable composite resin and Mineral Trioxide Aggregate (MTA) was used to fill the resorptive defect, and the coronal access was temporarily sealed. Composite restoration was subsequently replaced with ceramic crown after 4 years. Radiographs at 1 and 4 years showed adequate repair of the resorption and endodontic success. Clinically and radiographically the tooth was asymptomatic, and no periodontal pocket was found after a 4-year followup.

## 1. Introduction

 Root resorption is the process of the removal of cementum and/or dentine through physiological or pathological activity of tooth resorbing cells, which may be called dentoclasts. There are two types of tooth resorption: internal and external resorption. External tooth resorption has been classified into four types based on clinical and histological features, namely, external surface resorption, external inflammatory root resorption, replacement resorption, and ankylosis.

Invasive cervical resorption (ICR) is a type of external inflammatory root resorption. It is a relatively distinct clinical entity, although its wide spectrum of clinical presentations has resulted in its designation by different names such as progressive intradental resorption and invasive resorption [1, 2]. It is defined as a localized resorptive process that commences on the surface of the root below the epithelial attachment and the coronal aspect of the supporting alveolar process, namely, the zone of the connective tissue attachment [3].

Injury and stimulation by sulcular microorganisms in the adjacent marginal tissues is one of the cause [3–5]. Traumatic injuries, orthodontic tooth movement, orthognathic and dentoalveolar surgery, periodontal treatment, and internal bleaching are suggested as predisposing factors for the invasive cervical resorption [3, 6–9].

 ICR may be a barely discernable radiolucency or dramatically evident on the Periapical radiograph. The lesions vary in the shape from well-delineated radiolucencies with irregular borders and sometimes assume as caries radiographically. 

As ICR is initiated apical to the epithelial attachment, it is most commonly seen in the cervical area of the tooth, but it can be present anywhere in the root [10]. In the early stages, it may be somewhat symmetrical, but the larger lesions tend to be asymmetrical [11]. 

Heithersay developed a clinical classification to provide a clinical guide in the assessment of cases of invasive cervical resorption [9]. Frank and Torabinejad identified three different classes of resorption defect based on the location of the portal of entry in the cementum supraosseus, crestal, and intraosseus [8].

Mineral trioxide aggregate (MTA) is a biocompatible cement, which has good sealing ability, and is moisture tolerant [12–14]. When MTA was used to seal perforations in the furcal area, it induced the repair of the periodontium and new cementum formation over the material [15, 16].

Early diagnosis, elimination of the resorption, and restorative management are the keys to a successful outcome. This case report describes the successful management of a maxillary central incisor with an invasive cervical resorption surgically.

## 2. Case Report

**Figure 1:**
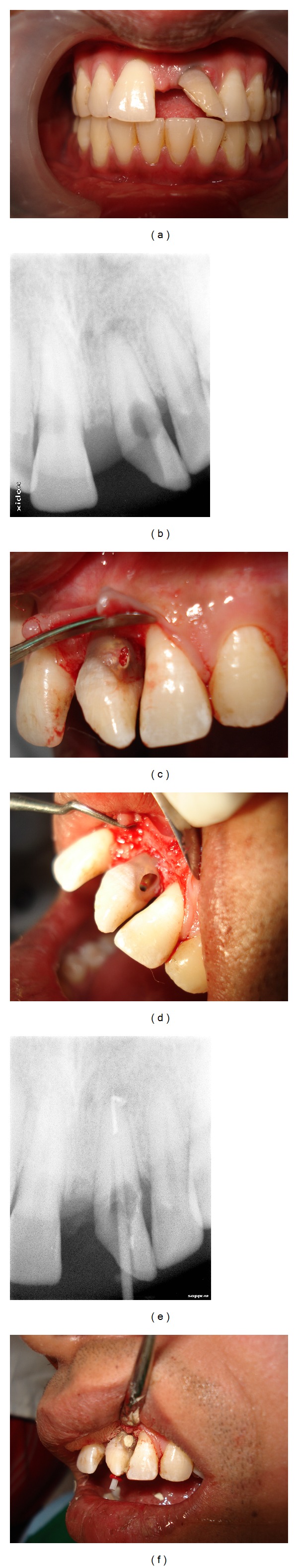
(a) Clinical photograph of case with fractured upper left central showing discolouration at the cervical area and sinus tract at distobuccal aspect. (b) Radiograph showing circular resorptive cavity which extends on the external aspect of distal buccal area. (c) Surgical site represents circular Resorptive site housed with the granulomatous tissue. (d) Clinical photograph showing the removal of granulomatous tissue. (e) Radiograph shows the sectional obturation and placement of fibre post. (f) Resorptive site filled with white MTA.

 A 19-year-old male patient was presented to the Department of Conservative Density and Endodontics, with a fractured upper left central incisor, discolorations at the cervical area, and gingival overgrowth at distal aspect. The patient has noticed color change and it increased over 3 to 4 month. After taking the past medical history of the patient, he had undergone trauma 8 years back with 21 and teeth remained untreated. The electric pulp test was negative. Periodontal probing depths were physiological at all sites except for the distobuccal surface where sinus tract and the necrosed material were present (Figure 1(a)). The radiographic examination revealed an irregular radiolucent area in the cervical third of the external root surface (Figure 1(b)). Periapical radiolucent lesion was detected. The clinical diagnosis was irreversible pulpitis with class 3 invasive cervical resorption. 

## 3. Management 

 Root canal treatment with debridement and restoration of the resorption lacuna surgically was the treatment of choice. Consent was obtained from the patient. Under local anesthesia access cavity was opened on the palatal surface; the root canal was cleaned with manual instruments and 5% NaOCl (Hyposept, UPS Hygienes Pvt. Ltd., Mumbai, India) irrigation. After the root canal system was debrided, it was rinsed with sterile water and dried with paper points. 

**Figure 2:**
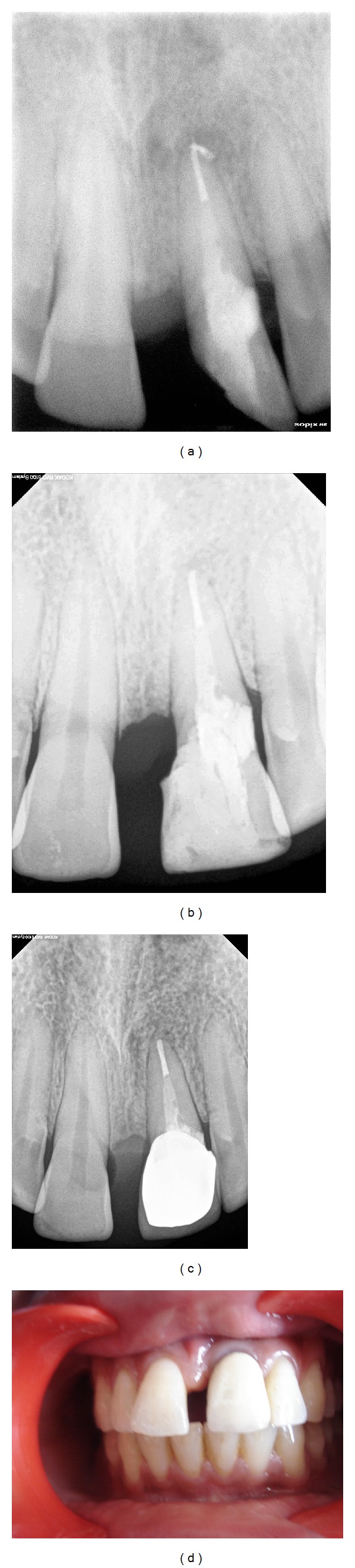
(a) Radiograph showing a complete root canal treatment. (b) 1-year followup showing no sign of Periapical pathosis. (c) 4-year followup radiograph. (d) 4-year followup clinical photograph.

 As the defect was on the cervical area, surgical intervention was planned. Incision was given and full thickness flap was reflected. A circular resorptive area was seen clearly from the surgical site housed with granulation tissue (Figure 1(c)). The resorptive area was cleaned by rinsing with alternating solutions of 5% NaOCl (Hyposept, UPS Hygienes Pvt. Ltd., Mumbai, India) and 17% EDTA (Prime Dental product Limited, India). Granulomatous tissue was removed from the surgical site efficiently. Then the residual caries were removed with the help of round bur no. 6 (MANI, burs, Japan). As the result the defect was enlarged in the size (Figure 1(d)). Sectional obturation was done with the help of 0.04% gutta percha (Dentsply, Maillefer, Switzerland). The post space was prepared with the help of Peeso Reamers (MANI. Peeso Reamers, Japan). The fibre post (3 M, ESPE, USA) was luted with the help of flowable composite resin material and then the resorptive area was filled with MTA (Proroot MTA, Dentsply, Mallifier, Switzerland) (Figures 1(e) and 1(f)) and the access cavity was temporarily sealed. After 3 days, the tooth was restored with dentine and enamel-bonded composite (Figure 2(a)). The patient subsequently received periodontal maintenance at 4-month intervals for 1 year. Clinical and radiographic examinations were performed at 12 and 48 months (Figures 2(b) and 2(c)). Repair of resorption defect was successfully treated with white MTA. Periodontal probing detected no pathological periodontal signs after 4 years (Figure 2(d)).

## 4. Discussion 

The basic aim of treating invasive cervical resorption is the complete removal of resorptive tissue and the restoration of the defect area. The present case describes a cervical resorptive defect in which tooth shows a sign of pulpal infection and requires root canal treatment followed by the post and core. The etiological factor for the case report is traumatic injury. The present case can be classified as a supraosseus defect or class 3 invasive cervical resorption; the communication between the resorption lacuna and the root canal system was large in size, and the defect was treated surgically [17].

ICR occurs immediately below the epithelial attachment of the tooth. As a result, it must be noticed that the location is not always cervical but related to the level of the marginal tissues and the pocket depth. Unless proper treatment is initiated, this type of resorption continues and a large irreversible loss of tooth structure may appear with time.

Root canal treatment and management of the resorption were performed in one session in order to avoid secondary infection [17]. During the debridement of the resorptive lacuna, the use of chemical escharotic agents, such as trichloroacetic acid (TCA), improves the possibility of completely eliminating resorbing cells, which penetrate into the deeper parts of the defect and enhance the visualization of the defect. In the case presented, the resorptive area was debrided with alternating solutions of 5% NaOCl and 17% EDTA (prime dental products Pvt. Ltd., Mumbai, India). The final rinse with sterile water was performed in the resorption site to enhance the adaptation of MTA [18].

In the recent literature reviews, it has become clear that posts do not strengthen endodontically treated teeth, and their use is justified only for the retention of the coronal restoration [19, 20].

 Tooth-colored fiber posts were introduced in the 1990s being and having several advantages, such as esthetic, bond to tooth structure, and having a modulus of elasticity similar to that of dentin, but still require dentin preparation to fit into the canal [21, 22]. 

When a tooth has more than 50% of its coronal structure missing, the use of a post-and-core foundation is recommended prior to restoration [23].

Mineral trioxide aggregate was chosen as the filling material for its biocompatibility and for its sealing ability [12, 13]. In previous studies, MTA was successfully used to repair communication between the pulp canal space and the periodontal tissue that occurs in cases of root perforation in dogs and humans [15, 24, 25].

This case show that ICR can be arrested using the “Heithersay approach” to treatment (i.e., mechanical debridement, treatment with trichloroacetic acid, and restoration). Prudent case selection and proper execution can lead to the successful treatment and long-term retention of the tooth. Although this case report presents a favourable outcome, further studies are encouraged to support the use of MTA to fill external invasive cervical resorption.
